# EGFR T790M relative mutation purity predicts osimertinib treatment efficacy in non-small cell lung cancer patients

**DOI:** 10.1186/s40169-020-0269-y

**Published:** 2020-02-17

**Authors:** Qiufan Zheng, Shaodong Hong, Yan Huang, Hongyun Zhao, Yunpeng Yang, Xue Hou, Yuanyuan Zhao, Yuxiang Ma, Ting Zhou, Yaxiong Zhang, Wenfeng Fang, Li Zhang

**Affiliations:** 1 Department of Medical Oncology, State Key Laboratory of Oncology in South China, Collaborative Innovation Center for Cancer Medicine, Sun Yat-Sen University Cancer Center, Guangzhou, 510060 Guangdong China; 2 Department of Clinical Research, State Key Laboratory of Oncology in South China, Collaborative Innovation Center for Cancer Medicine, Sun Yat-Sen University Cancer Center, Guangzhou, 510060 Guangdong China

**Keywords:** T790M mutation, Allele frequency, Osimertinib, Non-small-cell lung cancer

## Abstract

**Background:**

Despite the impressive anti-tumor activity of osimertinib in epidermal growth factor receptor (EGFR) T790M-positive non-small cell lung cancer (NSCLC) patients, 30–40% of patients still show limited response. There is therefore a need to identify biomarkers that accurately predict the response to osimertinib therapy. In this study, 54 patients with targeted next-generation sequencing of circulating tumor DNA before osimertinib treatment and known T790M positivity were included. We investigated the predictive value of baseline circulating tumor DNA-derived biomarkers on osimertinib therapy.

**Results:**

Baseline maximum somatic allele frequency (MSAF) level was not associated with objective response rate (ORR) (*P* = 0.886) and progression-free survival (PFS) (*P* = 0.370) of osimertinib treatment. T790M relative mutation purity (RMP, defined here as the ratio of T790M AF to MSAF) quartiles were found to be significantly associated with ORR (*P* for trend = 0.002) and PFS (*P* for trend = 0.006), and a cut off value of 0.24 identified two distinct prognostic groups [Hazard ratio (HR) = 0.36 for low T790M RMP, 95% confidence interval (CI) 0.18–0.72, *P* = 0.004). Additionally, although T790M relative mutation abundance (RMA, defined as T790M AF/EGFR driver AF) quartiles were not significantly associated with ORR (*P* for trend = 0.063), a cut off value of 0.30 also identified two distinct prognostic groups (HR = 0.43 for low T790M RMA, 95% CI 0.22–0.85, *P* = 0.015). However, in multivariate analysis, grouping of T790M RMP showed a better predictive value (HR = 0.46, 95% CI 0.20–1.05, *P* = 0.066) than T790M RMA (HR = 0.71, 95% CI 0.31–1.61, *P* = 0.409). Moreover, T790M RMP as continuous covariate was independently predictive of PFS (HR = 0.15, 95% CI 0.03–0.79, *P *=0.025), while T790M RMA was not (HR = 1.14, 95% CI 0.49–2.66, *P *=0.766). An external validation cohort further confirmed the T790M RMP was significantly associated with PFS of osimertinib therapy.

**Conclusions:**

This study established the independent predictive role of T790M RMP in NSCLC patients receiving osimertinib treatment.

## Background

The third-generation epidermal growth factor receptor (EGFR) tyrosine kinase inhibitors (TKI) osimertinib is the current standard of care for patients with advanced EGFR-positive non-small cell lung cancer (NSCLC) who acquired T790M mutation after receiving earlier-generation TKIs therapy [1]. Osimertinib significantly improves objective response rate (ORR) and progression-free survival (PFS) in NSCLC patients compared with chemotherapy of platinum-pemetrexed [2]. Despite the impressive anti-tumor activity of osimertinib, 30–40% of patients still show limited response [2–4].

There is therefore an urgent need to identify biomarkers that accurately predict for treatment response in NSCLC patients receiving osimertinib therapy. Currently, the proportion of T790M-positive clones within patient tumors may serve as a predictive biomarker for osimertinib treatment outcomes. Some prior studies have indicated that quantification of the T790M relative mutation abundance (RMA), which calculated as T790M allelic fraction (AF)/EGFR driver AF, is associated with the efficacy of third-generation EGFR TKIs [5–10]. However, none of the studies suggested the T790M RMA to be an independent predictor for response to osimertinib. Additionally, increasing evidence has demonstrated that EGFR-mutant NSCLC is not a single-oncogene disease [11, 12], and both EGFR-mutated and wild-type cancer cells can exist in the same tumor [13–15]. Considering that not every cancer cell harbors the EGFR driver mutation, the T790M RMA value does not accurately represent the proportion of T790M mutant cells in a given patient.

Circulating tumor DNA (ctDNA) is shed from tumor cells throughout the body. The maximum AF among all somatic mutations detected in plasma sample can provide an estimate of the ctDNA fraction in blood [16–18]. This may be based on the hypothesis that mutation with maximum AF in blood is shared by the most malignant cells and represents the largest clone in a given patient’s tumors. In the present study, we defined “the T790M relative mutation purity (RMP)” as the ratio of T790M AF to maximum somatic allele frequency (MSAF), and hypothesized that the T790M relative purity at baseline blood sample is more representative of the real proportion of T790M-mutant clone and could preferably predict the efficacy of osimertinib therapy.

## Methods

### Patients and data collection

This study was approved by the Institutional Review Board of Sun Yat-sen University Cancer Center. A total of 243 consecutive patients with advanced NSCLC performed blood-based next-generation sequencing (NGS) panel testing at initial diagnosis or disease progression between April 2017 and April 2019 were screened. We identified 54 patients who had T790M mutation detected and treated with osimertinib subsequently. Patient characteristics were obtained from retrospective electronic medical records. Efficacy was evaluated based on Response Evaluation Criteria In Solid Tumors (RECIST) v1.1 [19]. PFS was measured from the first day of osimertinib treatment to tumor progression or death date. ORR was defined as the percentage of patients with complete or partial response.

### Genomic profiling of ctDNA

All pre-osimertinib ctDNA samples were analyzed with College of American Pathologists (CAP)/Clinical Laboratory Improvement Amendments (CLIA)-certified genotyping assays (Additional file 1: Table S1). Testing methodologies included the BGI NGS platform and OrigiMed NGS platform [20, 21]. Ten milliliters of peripheral whole blood was collected in Strek tubes for genomic profiling of ctDNA. DNA extraction, library construction, and high-throughput sequencing was performed using the commercial panels of BGI OseqT containing 206 genes or its upgrading panel containing 513 genes [20], or OrigiMed Qiyuan panel containing 329 genes [21]. Results were analyzed for alterations including substitutions, short insertions/deletions, rearrangements, and copy number amplification.

### Determination of T790M RMA and T790M RMP

AF of each somatic mutation was calculated as the percentage of mutant DNA allele reads relative to total DNA allele reads (mutant plus wild type). We first corrected AF of each mutation by the copy numbers of the same gene with copy number gains, as described by Blakely and colleagues [12]. MSAF is determined by calculating the AF for all known somatic, likely somatic, and variant of unknown significance alterations, excluding those alterations that are likely germline. The MSAF value for each individual patient was the highest AF of the detected somatic variants in corresponding blood sample. The T790M RMA was calculated as the ratio of T790M AF to EGFR driver AF, according to previous reports [5–10, 22–24]. In patients where EGFR driver mutation was undetected, we assumed the ratio to be 1.0. For the calculation of T790M RMP, we identified the maximum corrected AF out of all alterations measured as the normalized MSAF; and calculated the ratio of the corrected AF of T790M over the normalized MSAF.

### Validation cohort

A public NSCLC cohort from Blakely et al. [12] of 34 patients (7 patients were excluded for T790M-negative) with T790M-mutant NSCLC treated with osimertinib was used for external validation. Patients in this cohort were tested on a ctDNA 68-gene panel (Guardant 360) before osimertinib treatment.

### Statistical analyses

The Wilcoxon signed-rank test, Mann–Whitney test, and χ^2^ test were used where applicable to compare clinical parameters and responses. *P* value for trend was obtained by regression analysis. Receiver operating characteristic (ROC) curves were generated to determine area under the curve (AUC) and optimal cut-off of T790M RMP and RMA for identifying patients with response. Survival curves were plotted using the Kaplan–Meier method and compared using the log-rank test. Hazard ratio (HR) and corresponding 95% confidence interval (CI) was determined through Cox proportional hazards regression analysis. Variables with a statistical significance of *P *≤ 0.10 in the univariate analysis were entered into the final multivariate model. Multivariate analysis was used to identify independent predictor associated with PFS.

## Results

### Patient characteristics

Patient characteristics are presented in Table 1. There were 35 women and 19 men with a median age of 58 years (range, 31–83 years). Twenty-six (48.1%) patients had brain metastases. Forty (74.1%) patients originally harbored EGFR exon 19 deletion and 14 (25.9%) harbored L858R mutation. All of the patients had detectable EGFR T790M mutation in ctDNA; however, one patient did not detect EGFR driver mutation at the baseline liquid biopsy. Three patients who had de novo T790M-positive tumors but received osimertinib as first-line treatment were also included. The median follow-up was 16.5 months (range, 3.4–30.1 months).

**Table 1:** Characteristics of included patients in our cohort

Characteristics	n (%)
Total	54 (100)
Age at start of osimertinib, years	
Median (range)	58 (31–83)
< 60	29 (53.7)
≥ 60	25 (46.3)
Sex	
Female	35 (64.8)
Male	19 (35.2)
Smoker	
Never	40 (74.1)
Ever	11 (20.4)
Unknown	3 (5.6)
Histology	
Adenocarcinoma	52 (96.3)
Adenosquamous carcinoma	2 (3.7)
Brain metastases	
Yes	26 (48.1)
None	28 (51.9)
Line of therapy	
1st/2nd^a^	38 (70.4)
3rd or more	16 (29.6)
NGS platform	
BGI^b^	29 (53.7)
OrigiMed	25 (46.3)
EGFR activating mutation	
Exon 19 deletion	40 (74.1)
Exon 21 L858R	14 (25.9)
TP53 status	
Mutated	33 (61.1)
Wild type	21 (38.9)

*NGS* next-generation sequencing, *EGFR* epidermal growth factor receptor

^a^Three patients had de novo T790M mutation and osimertinib were the first-line therapy

^b^Two samples analyzed with an upgrading BGI OseqT NGS panel

### Association between MSAF and osimertinib treatment outcomes

**Fig. 1:**
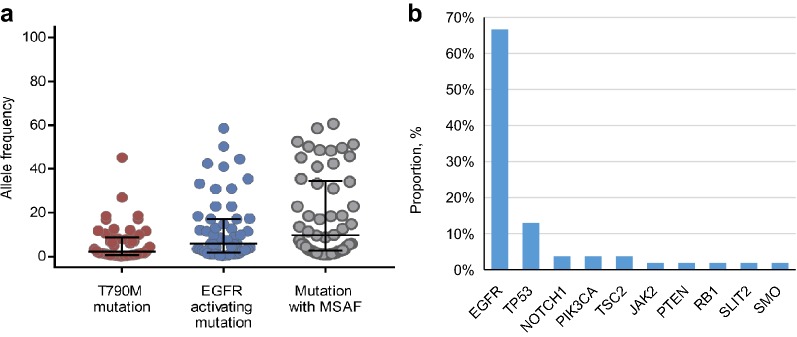
Maximum somatic allele frequency (MSAF). **a** Allele frequency of T790M mutation, EGFR activating mutation, and maximum somatic mutation in 54 patients. AFs were corrected with their copy numbers. **b** Distribution of the genes with maximum allele frequency among 54 patients. AFs were corrected with their copy numbers

**Fig. 2:**
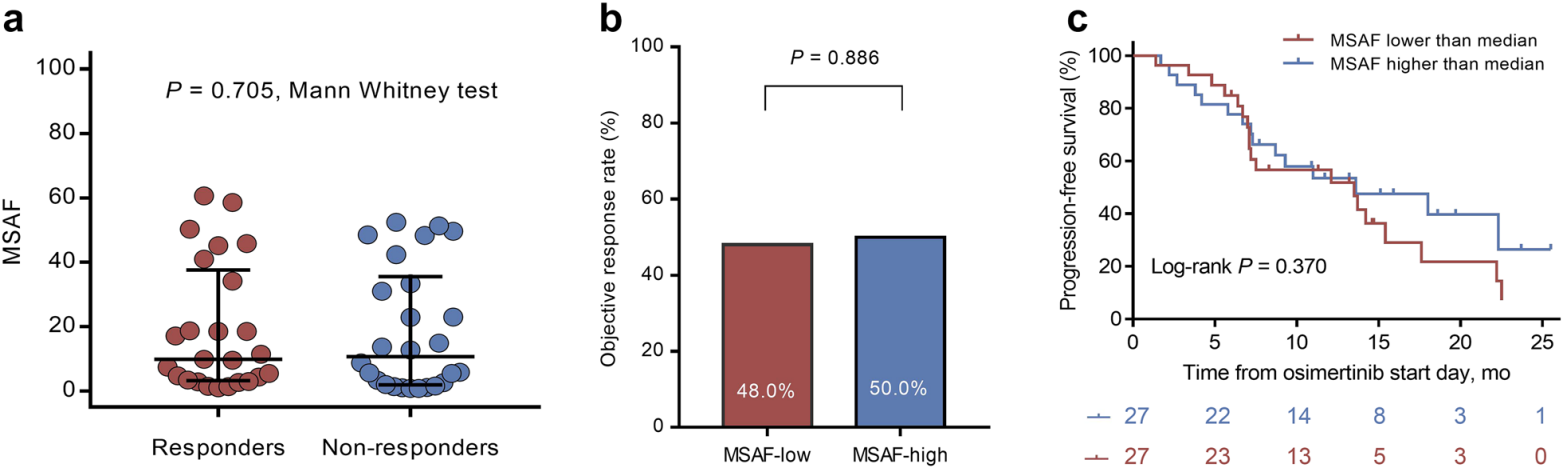
Maximum somatic allele frequency (MSAF) and osimertinib treatment outcomes. **a** The MSAF level between responders and non-responders (median: 9.9% vs 10.7%, *P* = 0.705, Mann–Whitney test). **b** Overall response rate between patients with MSAF-low and MSAF-high (48.0% vs 50.0%, *P* = 0.886, χ^2^ test), according to the median value of MSAF. **c** Progression-free survival stratified by MSAF-low and MSAF-high patients (*P* = 0.370, log-rank test)

**Fig. 3:**
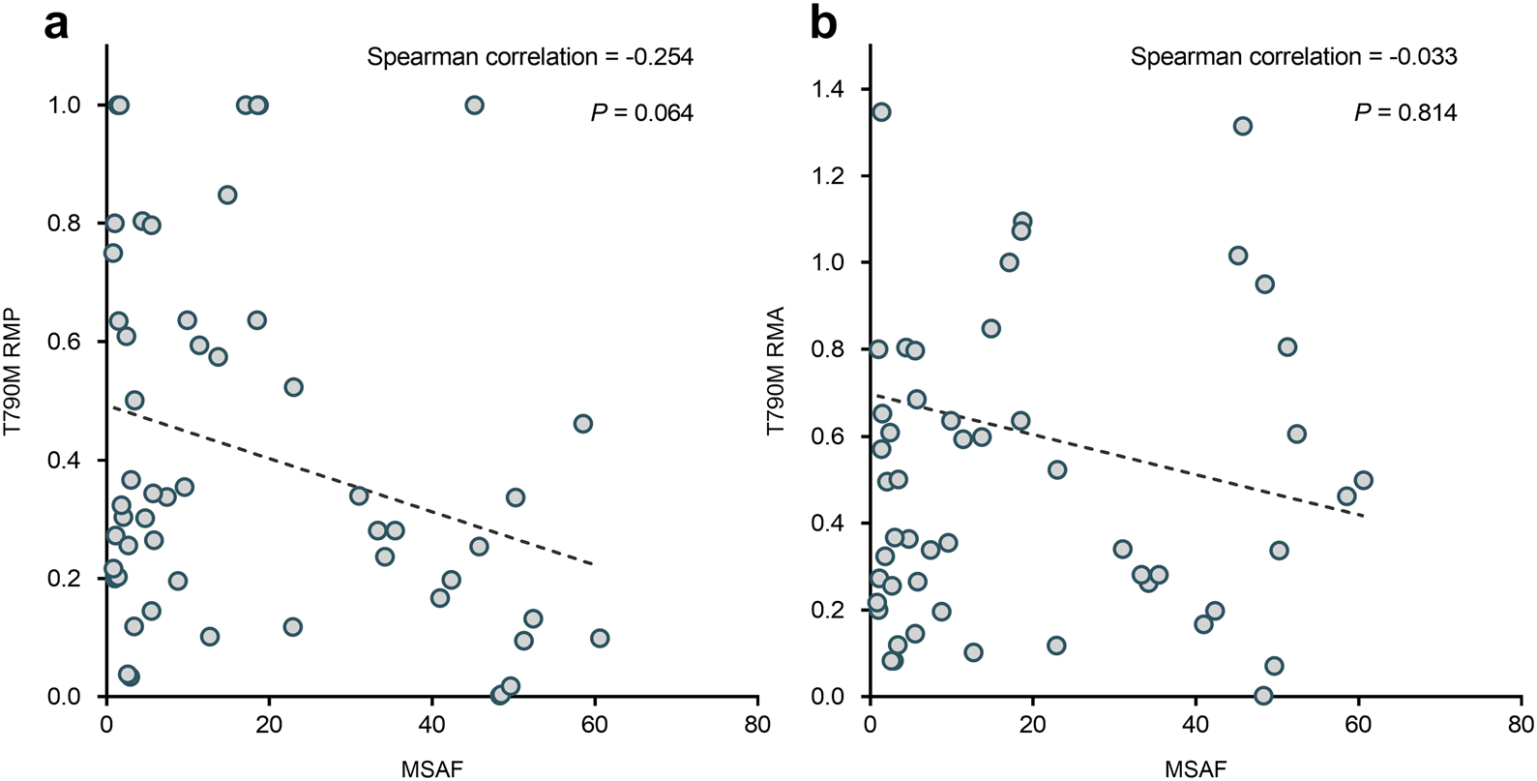
Maximum somatic allele frequency (MSAF) was not correlated with T790M relative mutation purity (RMP) and T790M relative mutation abundance (RMA). **a** Correlation between MSAF and T790M RMP in the 54 patients (Spearman correlation = − 0.254; *P* = 0.064). **b** Correlation between MSAF and T790M RMA in the 54 patients (Spearman correlation = − 0.033; *P* = 0.814)

At baseline, the median EGFR driver AF (5.7%, range = 0.2–58.5%) was significantly higher than T790M AF (2.2%, range = 0.1–45.2%; *P *< 0.001), while significantly lower than the median MSAF (9.73%, range = 0.8–60.6%; *P *< 0.001) (Fig. 1a). EGFR (66.7%) was the most frequently detected gene with maximum AF among all patients, following by TP53 (12.9%), PIK3CA (3.7%), NOTCH1 (3.7%), and TSC2 (3.7%) (Fig. 1b). Given that the ctDNA MSAF level have been found to be a prognostic factor in previous reports [21, 25–30], we first compared the MSAF level between responders and non-responders. However, we did not find a significant difference between them in our study (*P *=0.705; Fig. 2a). We further divided patients into two groups (MSAF-high and MSAF-low) according to the value of median MSAF. However, we still did not observe any differences in response and PFS between two groups (Fig. 2b, c). In addition, both T790M RMP and RMA were not correlated with MSAF (Fig. 3a, b).

### Association between T790M RMP and osimertinib treatment outcomes

**Fig. 4:**
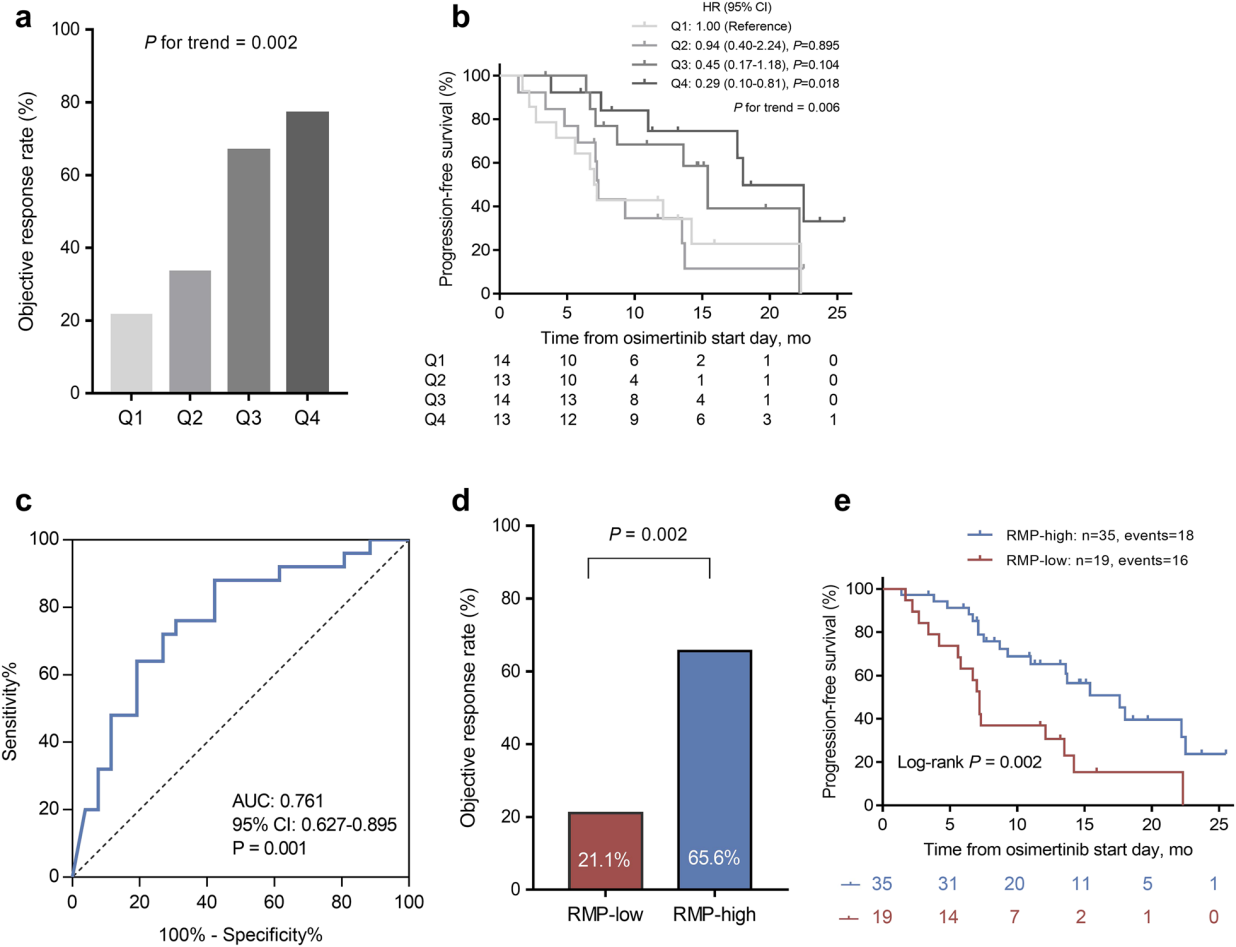
Correlation between T790M relative mutation purity (RMP) and osimertinib treatment outcomes. **a** Objective response rate (ORR) stratified by T790M RMP quartiles (*P* for trend = 0.002). **b** Progression-free survival (PFS) stratified by T790M RMP quartiles (*P* for trend = 0.006). **c** Receiver Operating Characteristic (ROC) Curve and area under ROC curve for T790M RMP predicting objective response. **d** ORR stratified by RMP-low and RMP-high patients (21.1% vs 65.6%, *P* = 0.002). **e** PFS stratified by RMP-low and RMP-high patients (P = 0.013, log-rank test)

To investigate the relationship between outcomes and T790M RMP level, we divided patients into four quartiles (Q1–Q4) according to the value of RMP. We compared the ORRs among four quartiles (Q1–Q4), and observed a significantly positive correlation between T790M RMP level and ORRs (ORR = 21.4%, 33.3%, 66.7%, and 76.9% for Q1 to Q4 of T790M RMP, respectively; *P* for trend = 0.002; Fig. 4a). We also observed a striking association for longer PFS with increasing quartiles of RMP (*P* for trend = 0.006). Median PFS was 7.0 months, 7.3 months, 15.4 months and 18.0 months for patients with Q1 to Q4 of T790M RMP, respectively (Fig. 4b). At the extremes, patients with the highest T790M RMP (Q4) had a 71% reduced risk of disease progression or death compared with patients with lowest T790M RMP (Q1) (HR = 0.29, 95% CI 0.10–0.81, *P *= 0.018). To facilitate practical application of our findings, we determined the optimal T790M RMP cut-off for response using a ROC-determined value of 0.24 (area under the curve = 0.761) (Fig. 4c). Above this cut-off (n = 35), patients presented an ORR of 65.6% and median PFS of 17.6 months, whereas below this cut-off (n = 19), patients presented an ORR of 21.1% (*P* = 0.002; Fig. 4d) and median PFS of 7.2 months (log-rank *P* = 0.002; Fig. 4e).

### Association between T790M RMA and osimertinib treatment outcomes

**Fig. 5:**
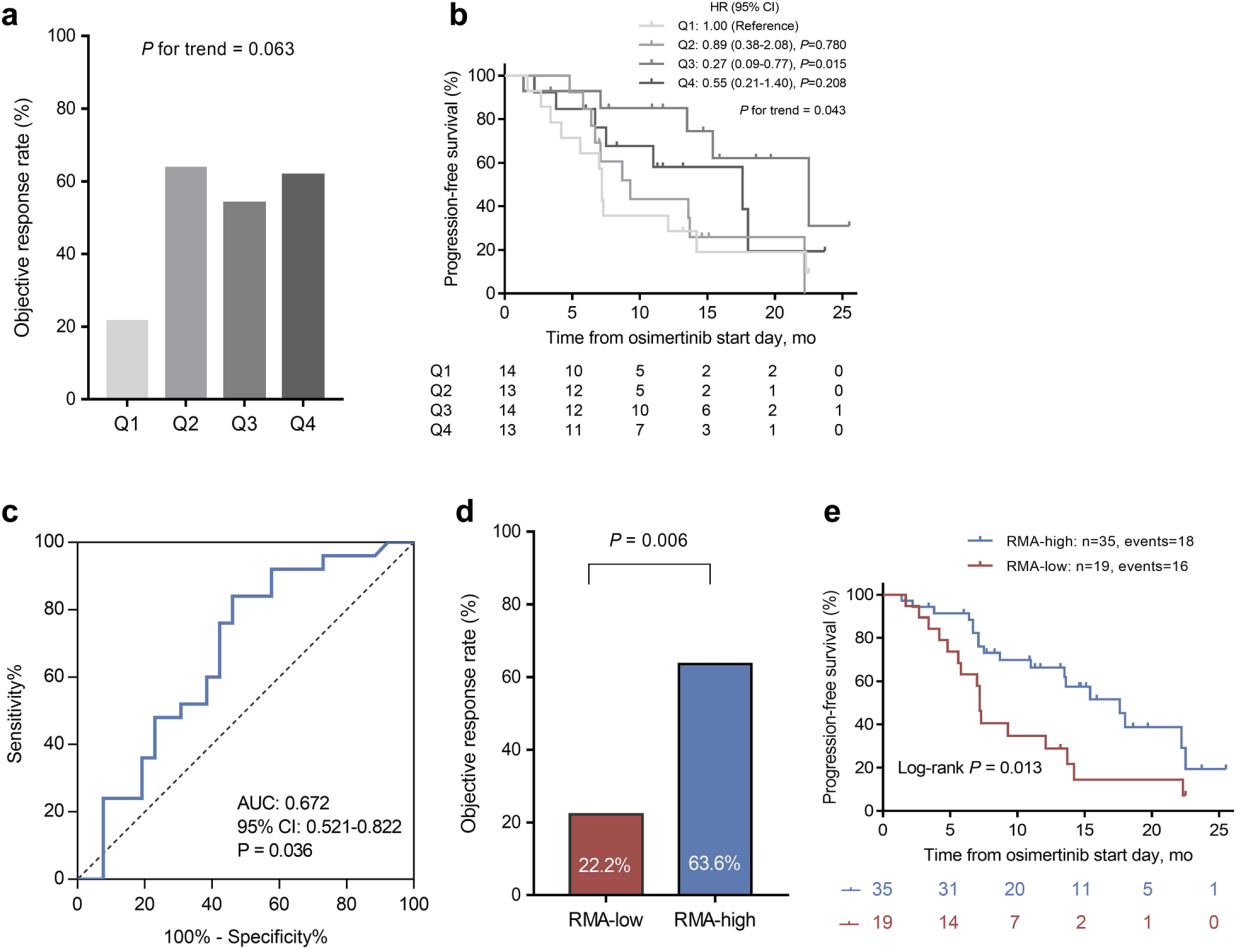
Correlation between T790M relative mutation abundance (RMA) and osimertinib treatment outcomes. **a** Objective response rate (ORR) stratified by T790M RMA quartiles (*P* for trend = 0.063). **b** Progression-free survival (PFS) stratified by T790M RMA quartiles (*P* for trend = 0.043). **c** Receiver Operating Characteristic (ROC) Curve and area under ROC curve for T790M RMA predicting objective response. **d** ORR stratified by RMA-low and RMA-high patients (22.2% vs 63.6%, *P* = 0.006). **e** PFS stratified by RMA-low and RMA-high patients (P = 0.013, log-rank test)

Similarly, we analyzed the T790M RMA, and found a positive association between T790M RMA quartiles and ORRs; however it was not statistically significant (*P* for trend = 0.063; Fig. 5a). And although there was a statistically significant association trend towards PFS (*P* for trend = 0.043), it is noteworthy that the third quartile (Q3) of RMA showed the greatest risk reduction in disease progression or death compared with lowest T790M RMA (Q1) (HR = 0.27, 95% CI 0.09–0.77; Fig. 5b). ROC analysis determined that the optimal RMA cut-off for response was 0.3 (Fig. 5c). Above this cut-off (n = 35), patients presented an ORR of 63.6% and median PFS of 17.6 months, whereas below this cut-off (n = 19), patients presented an ORR of 22.2% (*P* = 0.006; Fig. 5d) and median PFS of 7.2 months (log-rank *P* = 0.013; Fig. 5e).

### Cox regression analysis identified T790M RMP as independent predictor

Further, we performed Cox regression analysis to determine whether T790M RMP or RMA is the better predictive biomarker in patients. Firstly, we analyzed relative T790M RMP and RMA as continuous covariates without bias produced by grouping in the univariate analysis. We found that T790M RMP was significantly associated with PFS (HR = 0.14, 95% CI 0.04–0.56, *P *=0.005), while T790M relative abundance was not (HR = 0.40, 95% CI 0.14–1.16, *P *=0.091) (Table 2). Moreover, T790M RMP remained an independent predictive biomarker for PFS in multivariate analysis (HR = 0.15, 95% CI 0.03–0.79, *P *=0.025) (Table 3). We then analyzed T790M RMP and RMA as categorical variables (both using the cut-off determined by ROC analysis). In univariate analyses, both T790M RMP and RMA were significantly associated with PFS (HR = 0.36, 95% CI  0.18–0.72, *P* = 0.004; and HR = 0.43, 95% CI 0.22–0.85, *P* = 0.015; respectively) (Table 2). However, in multivariate analysis, T790M RMP was confirmed to be a better predictive biomarker (HR = 0.46, 95% CI 0.20–1.05, *P* = 0.066) than T790M RMA (HR = 0.71, 95% CI 0.31–1.61, *P* = 0.409) (Table 3).

**Table 2:** Univariate analysis for progression free survival in our cohort

Characteristics	Univariate analyses	
	HR (95% CI)	*P* value
Age at start of osimertinib, years		
< 60	1.00 (Reference)	
≥ 60	0.75 (0.38–1.51)	0.425
Sex		
Female	1.00 (Reference)	
Male	1.24 (0.62–2.47)	0.548
Smoker		
Never	1.00 (Reference)	
Ever	1.40 (0.64–3.07)	0.401
Unknown	1.51 (0.19–11.80)	0.692
Brain metastases		
Yes	1.00 (Reference)	
None	0.72 (0.36–1.43)	0.345
Line of therapy		
1st/2nd^a^	1.00 (Reference)	
3rd or more	1.05 (0.49–2.24)	0.900
NGS platform		
BGI^b^	1.00 (Reference)	
OrigiMed	1.19 (0.61–2.34)	0.612
EGFR activating mutation		
Exon 19 deletion	1.00 (Reference)	
Exon 21 L858R	1.21 (0.57–2.56)	0.618
MSAF		
Lower than the median MSAF	1.00 (Reference)	
Higher than the median MSAF	0.73 (0.37–1.45)	0.373
TP53 status		
Mutated	1.00 (Reference)	
Wild type	0.53 (0.25–1.11)	0.092
T790M RMA (continuous)	0.40 (0.14–1.16)	0.091
T790M RMP (continuous)	0.14 (0.04–0.56)	0.005
T790M RMA (categorical)		
≤ 0.30	1.00 (Reference)	
> 0.30	0.43 (0.22–0.85)	0.015
T790M RMP (categorical)		
≤ 0.24	1.00 (Reference)	
> 0.24	0.36 (0.18–0.72)	0.004

*HR* hazard ratio, *CI* confidence interval, *NGS* next-generation sequencing, *EGFR* epidermal growth factor receptor, *MSAF* maximum somatic allele frequency, *RMA* Relative mutation abundance, *RMP* Relative mutation purity

^a^Three patients had de novo T790M mutation and osimertinib were the first-line therapy

^b^Two samples analyzed with an upgrading BGI OseqT NGS panel

**Table 3:** Multivariate analysis for progression free survival in our cohort

Characteristics	Multivariate analyses	
	HR (95% CI)	*P* value
Model 1		
TP53 status		
Mutated	1.00 (Reference)	
Wild type	0.66 (0.31–1.43)	0.298
T790M RMA (continuous)	1.14 (0.49–2.66)	0.766
T790M RMP (continuous)	0.15 (0.03–0.79)	0.025
Model 2		
TP53 status		
Mutated	1.00 (Reference)	
Wild type	0.58 (0.28–1.24)	0.161
T790M RMA (categorical)		
≤ 0.30	1.00 (Reference)	
> 0.30	0.71 (0.31–1.61)	0.409
T790M RMP (categorical)		
≤ 0.24	1.00 (Reference)	
> 0.24	0.46 (0.20–1.05)	0.066

*HR* hazard ratio, *CI* confidence interval, *RMA* Relative mutation abundance, *RMP* Relative mutation purity

### Validation cohort confirmed our findings

In validation cohort (n = 34), T790M RMP as a continuous covariate was also significantly associated with PFS (HR = 0.16, 95% CI 0.03–0.87, *P* = 0.034), but T790M RMA was not (HR = 0.27, 95% CI 0.07–1.03, *P* = 0.056). In addition, T790M RMP as a dichotomous variable was also found to be significantly associated with PFS (HR = 0.35, 95% CI 0.13–0.93, *P* = 0.035), but the level of T790M RMA was still not (HR = 0.72, 95% CI 0.29–1.78, *P *=0.475).

### Subgroup analysis

**Fig. 6:**
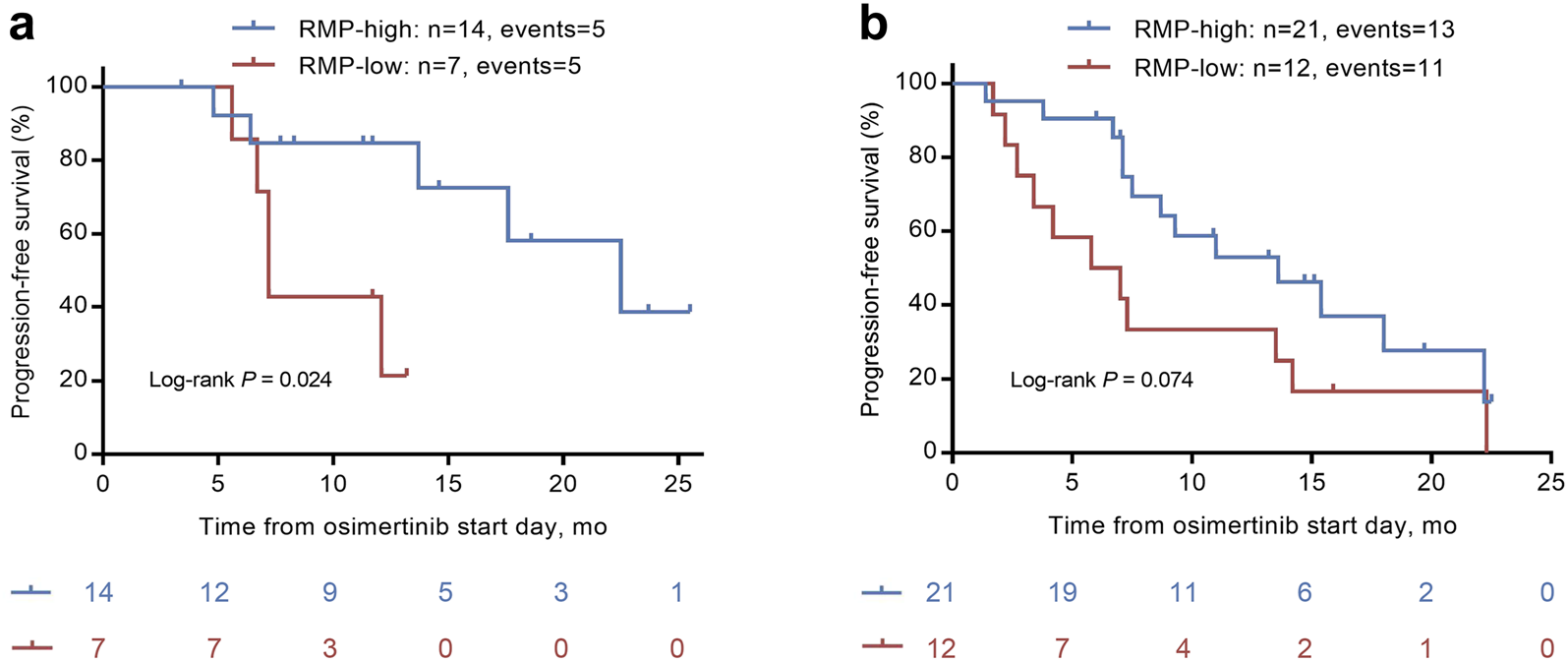
Subgroup analysis according to TP53 status. **a** Progression-free survival (PFS) stratified by T790M relative mutation purity (RMP)-low and RMP-high patients in TP53 wild-type population (*P* = 0.024, log-rank test). **b** PFS stratified by T790M RMP-low and RMP-high patients in TP53 mutated population (*P* = 0.074, log-rank test)

Concurrent mutations are well-established prognostic factors for EGFR TKI treatment in NSCLC, where TP53 mutation is the most common concurrent mutation [11, 12]. We finally performed subgroup analysis to assess the predictive value of T790M RMP in patients whose tumors presented with or without TP53 mutation. We found that no matter in TP53 mutated or TP53 wild-type population, patients with high T790M RMP both had a longer PFS than those with low T790M RMP (Fig. 6a, b).

## Discussion

This study shows that a high level of T790M RMP, defined here as the ratio of T790M AF to MSAF value at baseline, is an independent predictor of prolonged PFS in T790M-positive NSCLC patients treated with osimertinib. Our findings suggested that T790M RMP was superior to T790M RMA in estimating proportion of T790M-positive clones within an individual patient’s tumors.

EGFR-mutant lung cancers will inevitably develop resistance after treatment with earlier-generation EGFR TKI. Multiple different resistance mechanisms can be developed within the same tumor specimen [31, 32], and different resistance mechanisms can also be developed in separate tumor deposits within the same patient. Thus, in addition to T790M-positive clones, T790M-negative clones harboring EGFR-dependent or EGFR-independent resistance mechanisms may also occur. However, osimertinib was only sensitive to T790M-positive clones, but poorly effective to T790M-negative clones [3]. Thus, investigating surrogates that can represent the proportion of T70M-positive cells and predict osimertinib efficacy is feasible and essential. There has been much interest in the analysis of surrogates, such as T790M AF [22, 24, 33–35], EGFR driver AF [7, 22], and T790M RMA level [5–10, 22–24] in plasma to predict response to osimertinib. However, inconsistent associations were observed across these studies. In the present study, increasing of T790M RMA did not show a significant association with prolonged PFS, both in our cohort and external validation cohort. Dichotomization of T790M RMA using a cut-off determined by ROC analysis was capable of predicting osimertinib treatment outcomes. However, PFS did not differ significantly between two groups in the multivariate analysis. In fact, in EGFR-mutant NSCLC, EGFR-driver clone is the largest clone in most but not all cases (e.g., 66.7% in our cohort). Baseline T790M RMA is a useful measure of potential response, but is not independent of baseline T790M RMP. This presumably reflects the T790M RMP is more correlated with the proportion of osimertinib-sensitive clones in patient tumors than the T790M RMA.

MSAF is a valid tool for quantifying the tumor fraction of cell free DNA [16–18]. Numerous studies have shown that the MSAF, or baseline ctDNA level, is a prognostic factor in patients receiving chemotherapy [25], targeted therapy [26, 27], and immunotherapy [21, 28–30], although most of the prognostic value of MSAF is correlated with baseline tumor burdens in patients. In one small size study (n = 11) [27], MSAF was also found to be associated with PFS in patients receiving osimertinib treatment. In contrast to their finding, we did not observed different treatment outcomes between high and low MSAF patients. However, we did observe that relative T790M purity, which represents the relative amount of T790M mutation in ctDNA, was significantly associated with osimertinib treatment outcomes, including ORR and PFS. The predictive value for T790M RMP was also confirmed in a validation cohort from an independent public data set.

Although sequencing of ctDNA is now routinely used in clinical practice, tissue-based genomic testing remains the gold standard to fully understand the heterogeneity of the mechanisms of resistance. However, sequencing of ctDNA can potentially provide a more representative profile of the overall predominant resistance mechanisms for a given patient than a core biopsy from one region of a single metastatic lesion. Moreover, with the restriction of intratumoral and intertumoral heterogeneity, tumor tissue-based sequencing might be unsuitable for providing prognostic parameters like T790M RMA and RMP. Our results further support the advantage of liquid biopsy over tumor biopsy sampling, which provides more informative predictors in the management of NSCLC patients treated with osimertinib.

Some limitations of this study need to be acknowledged. There is a subset of patients who were tumor T790M positive while plasma T790M negative, and the T790M RMP level is useless in this population. We used different NGS panel data in our study rather than a single central panel. The different panels may have varied analysis pipelines. However, we did not find the different panels were associated with MSAF or treatment outcome, and an independent validation cohort using the other NGS panel also confirmed our findings, suggesting the effect of different panels might be small. In addition, we only analyzed the impact of TP53 mutation status on treatment outcomes, while other co-occurring mutations were not analyzed, though others were rare events. Moreover, the limited sample size, the retrospective nature, and the single-centered design might yield statistical bias. Further large prospective multicentre validation with uniform NGS platform is needed to confirm our findings.

## Conclusion

We demonstrated that T790M RMP is a surrogate marker for proportion of T790M-positive clone in NSCLC patients. In this study established the independent predictive role of T790M RMP in NSCLC patients receiving osimertinib treatment, where higher T790M RMP predicts superior ORR and PFS.

## Supplementary information

**Additional file 1: Table S1.** Gene lists of targeted next-generation sequencing panels our study involved.

## Data Availability

The datasets used and/or analyzed during the current study are available from the corresponding author on reasonable request.

## Abbreviations

EGFR: Epidermal growth factor receptor
TKI: Tyrosine kinase inhibitor
NSCLC: Non-small cell lung cancer
ORR: Objective response rate
PFS: Progression-free survival
RMA: Relative mutation abundance
AF: Allelic frequency
ctDNA: Circulating tumor DNA
RMP: Relative mutation purity
MSAF: Maximum somatic allele frequency
NGS: Next-generation sequencing
CAP: College of American pathologists
CLIA: Clinical laboratory improvement amendments
ROC: Receiver operating characteristic
AUC: Area under the curve
HR: Hazard ratio
CI: Confidence interval
