# Supplementary material for: EGFR T790M relative mutation purity predicts osimertinib treatment efficacy in non-small cell lung cancer patients
Source: Clin Transl Med. 2020 Feb 17;9:17. doi: 10.1186/s40169-020-0269-y (PMC7026329; doi:10.1186/s40169-020-0269-y)
Supplement: Supplementary file 1 — Additional file 1: Table S1. Gene lists of targeted next-generation sequencing panels our study involved. [file 40169_2020_269_MOESM1_ESM.doc]

Additional file 1: Table S1. Gene lists of targeted next-generation sequencing panels our study involved.

| **BGI OseqTM panel (206 genes)** | | | | | | | | |
| --- | --- | --- | --- | --- | --- | --- | --- | --- |
| *ABL1* | *C11orf30* | *EPHA2* | *FUS* | *KIF5B* | *NF1* | *PPARG* | *SND1* | |
| *ACVRL1* | *CBL* | *EPHA3* | *GAB2* | *KIT* | *NF2* | *PPP2R1A* | *SOX2* | |
| *AKT1* | *CBR3* | *ERBB2* | *GATA3* | *KRAS* | *NFIB* | *PRCC* | *SPOP* | |
| *AKT2* | *CCDC6* | *ERBB3* | *GNA11* | *MAML2* | *NOTCH1* | *PRKAA1* | *SRC* | |
| *AKT3* | *CCND1* | *ERBB4* | *GNAQ* | *MAP2K1* | *NOTCH2* | *PSMB5* | *STAT3* | |
| *ALK* | *CCND2* | *ERG* | *GNAS* | *MAPK1* | *NOTCH3* | *PTCH1* | *STK11* | |
| *APC* | *CCNE1* | *ESR1* | *HDAC1* | *MAX* | *NOTCH4* | *PTEN* | *SUZ12* | |
| *AR* | *CD44* | *ETV6* | *HDAC2* | *MCL1* | *NR4A3* | *PTPN11* | *TAF15* | |
| *ARAF* | *CD74* | *EWSR1* | *HDAC3* | *MDM2* | *NRAS* | *RAC1* | *TCF3* | |
| *ASPSCR1* | *CDH1* | *EZH2* | *HDAC4* | *MDM4* | *NTRK3* | *RAF1* | *TERT* | |
| *ATF1* | *CDK4* | *EZR* | *HDAC6* | *MED12* | *NUTM1* | *RANKL* | *TET2* | |
| *ATM* | *CDK6* | *FBXW7* | *HDAC8* | *MET* | *PARP1* | *RB1* | *TFE3* | |
| *ATP11B* | *CDKN2A* | *FCGR2A* | *HGF* | *MLH1* | *PARP2* | *RET* | *TMPRSS2* | |
| *ATR* | *CDKN2B* | *FCGR2B* | *HNF1A* | *MLH3* | *PAX5* | *RHEB* | *TP53* | |
| *AURKA* | *CHEK2* | *FCGR3A* | *HRAS* | *MPL* | *PAX8* | *RHOA* | *TPM3* | |
| *BAP1* | *CREB1* | *FGD4* | *IDH1* | *MS4A1* | *PBX1* | *RICTOR* | *TRIM33* | |
| *BCL2* | *CRTC1* | *FGFR1* | *IDH2* | *MSH2* | *PD-1* | *ROS1* | *TSC1* | |
| *BCL2L1* | *CSF1R* | *FGFR2* | *IGF1R* | *MSH3* | *PDGFRA* | *RPS6KB1* | *TSC2* | |
| *BCR* | *CSNK2A1* | *FGFR3* | *IL6* | *MSH6* | *PDGFRB* | *SF3B1* | *U2AF1* | |
| *BIRC2* | *CTLA4* | *FGFR4* | *IRS2* | *MTOR* | *PD-L1* | *SLC34A2* | *VEGFA* | |
| *BIRC3* | *CTNNB1* | *FLCN* | *JAK1* | *MYB* | *PIK3CA* | *SLC45A3* | *VHL* | |
| *BRAF* | *DDR1* | *FLI1* | *JAK2* | *MYC* | *PIK3CB* | *SMAD2* | *WT1* | |
| *BRCA1* | *DDR2* | *FLT1* | *JAK3* | *MYCN* | *PIK3R1* | *SMAD4* | *XPO1* | |
| *BRCA2* | *DNMT3A* | *FLT3* | *JAZF1* | *MYD88* | *PLAG1* | *SMARCA4* | *ZNF217* | |
| *BRD4* | *EGFR* | *FLT4* | *KDR* | *NCOA4* | *PMS1* | *SMARCB1* |  | |
| *BTK* | *EML4* | *FOXL2* | *KIAA1549* | *NDRG1* | *POU5F1* | *SMO* |  | |
| **BGI OseqTM panel (513 genes)** | | | | | | | | |
| *ABL1* | *CBR1* | *EPCAM* | *GATA1* | *KMT2A* | *NPM1* | *RAD52* | *STAT3* | |
| *ABL2* | *CCND1* | *EPHA2* | *GATA2* | *KMT2B* | *NR3C1* | *RAF1* | *STAT4* | |
| *ACVR1B* | *CCND2* | *EPHA3* | *GATA3* | *KMT2C* | *NRAS* | *RARA* | *STAT5B* | |
| *ACVR2A* | *CCND3* | *EPHA5* | *GNA11* | *KMT2D* | *NSD1* | *RARB* | *STK11* | |
| *ACVRL1/ALK1* | *CCNE1* | *EPHB1* | *GNA13* | *KNG1* | *NTRK1* | *RARG* | *SUFU* | |
| *AKT1* | *CD79A* | *EPHB2* | *GNAQ* | *KRAS* | *NTRK2* | *RB1* | *SUZ12* | |
| *AKT2* | *CD79B* | *EPHB6* | *GNAS* | *LCK* | *NTRK3* | *REL* | *SYK* | |
| *AKT3* | *CDC25C* | *EPPK1* | *GNRHR* | *LIMK1* | *NUP93* | *RET* | *TAF1* | |
| *ALK* | *CDC42* | *ERBB2* | *GPR124* | *LRRK2* | *OTOS* | *RFC1* | *TBL1XR1* | |
| *ALOX12B* | *CDC73* | *ERBB3* | *GRIN2A* | *LYN* | *PAK3* | *RHEB* | *TBX3* | |
| *AMER1* | *CDH1* | *ERBB4* | *GRM3* | *MAP2K1* | *PAK7* | *RICTOR* | *TEK* | |
| *ANGPT1* | *CDK12* | *ERCC1* | *H3F3A* | *MAP2K2* | *PALB2* | *RNASEL* | *TERT* |  |
| *ANGPT2* | *CDK2* | *ERCC2* | *H3F3C* | *MAP2K4* | *PARP1* | *RNF43* | *TET2* |  |
| *APC* | *CDK4* | *ERCC3* | *HCK* | *MAP3K1* | *PARP2* | *ROBO1* | *TFG* |  |
| *APCDD1* | *CDK6* | *ERG* | *HDAC1* | *MAP3K13* | *PARP3* | *ROBO2* | *TGFBR2* |  |
| *AR* | *CDK8* | *ESR1* | *HDAC2* | *MAPK1* | *PARP4* | *ROS1* | *TIPARP* |  |
| *ARAF* | *CDKN1A* | *ETV1* | *HDAC3* | *MAPK3* | *PAX5* | *RPL22* | *TLR4* |  |
| *ARFRP1* | *CDKN1B* | *ETV6* | *HDAC4* | *MAPK8* | *PAX8* | *RPL5* | *TMEM127* |  |
| *ARHGAP35* | *CDKN2A* | *EWSR1* | *HDAC6* | *MAPK8IP1* | *PBRM1* | *RPS14* | *TNFAIP3* |  |
| *ARID1A* | *CDKN2B* | *EXT1* | *HDAC8* | *MAX* | *PCBP1* | *RPS6KB1* | *TNFRSF14* |  |
| *ARID1B* | *CDKN2C* | *EXT2* | *HGF* | *MC1R* | *PCM1* | *RPTOR* | *TNFRSF8* |  |
| *ARID2* | *CDX2* | *EZH2* | *HIF1A* | *MCL1* | *PD1/PDCD1* | *RUNX1* | *TNFSF11/RANKL* |  |
| *ARID5B* | *CEBPA* | *FAM46C* | *HIST1H1C* | *MDM2* | *PDGFRA* | *RUNX1T1* | *TNFSF13B* |  |
| *ASXL1* | *CFLAR* | *FANCA* | *HIST1H2BD* | *MDM4* | *PDGFRB* | *RXRA* | *TOP1* |  |
| *ATM* | *CHD1* | *FANCC* | *HIST1H3B* | *MECOM* | *PDK1* | *RXRB* | *TOP2A* |  |
| *ATR* | *CHD2* | *FANCD2* | *HNF1A* | *MED12* | *PDL1/CD274* | *RXRG* | *TOP2B* |  |
| *ATRX* | *CHD4* | *FANCE* | *HRAS* | *MEF2B* | *PHF6* | *SDHAF2* | *TP53* |  |
| *AURKA* | *CHEK1* | *FANCF* | *HRH2* | *MEN1* | *PIGF* | *SDHB* | *TRAF7* |  |
| *AURKB* | *CHEK2* | *FANCG* | *HSD17B3* | *MET* | *PIK3C2A* | *SDHC* | *TSC1* |  |
| *AXIN1* | *CHUK* | *FANCI* | *HSD3B2* | *MITF* | *PIK3C2B* | *SDHD* | *TSC2* |  |
| *AXIN2* | *CIC* | *FANCL* | *HSP90AA1* | *MLH1* | *PIK3C2G* | *SEMA3A* | *TSHR* |  |
| *AXL* | *CREBBP* | *FANCM* | *HSPA4* | *MLH3* | *PIK3C3* | *SEMA3E* | *TSHZ2* |  |
| *B2M* | *CRIPAK* | *FAT3* | *IDH1* | *MPL* | *PIK3CA* | *SETBP1* | *TSHZ3* |  |
| *B4GALT3* | *CRKL* | *FBXW7* | *IDH2* | *MRE11A* | *PIK3CB* | *SETD2* | *TUBA1A* |  |
| *BACH1* | *CRLF2* | *FCGR1A* | *IFNAR1* | *MS4A1* | *PIK3CG* | *SF1* | *TUBB* |  |
| *BAK1* | *CROT* | *FCGR2A* | *IFNAR2* | *MSH2* | *PIK3R1* | *SF3B1* | *TUBD1* |  |
| *BAP1* | *CSF1R* | *FCGR2B* | *IGF1* | *MSH3* | *PLK1* | *SH2B3* | *TUBE1* |  |
| *BARD1* | *CTCF* | *FCGR3A* | *IGF1R* | *MSH4* | *PML* | *SIN3A* | *TUBG1* |  |
| *BCL2* | *CTLA4* | *FCGR3B* | *IGF2* | *MSH5* | *PMS1* | *SLAMF7* | *TYR* |  |
| *BCL2A1* | *CTNNA1* | *FGF10* | *IKBKB* | *MSH6* | *PMS2* | *SLC4A1* | *U2AF1* |  |
| *BCL2L1* | *CTNNB1* | *FGF12* | *IKBKE* | *MSR1* | *POLQ* | *SLIT2* | *UMPS* |  |
| *BCL2L11* | *CUL4B* | *FGF14* | *IKZF1* | *MTOR* | *PPP2R1A* | *SMAD2* | *VEGFA* |  |
| *BCL2L2* | *CYLD* | *FGF19* | *IL7R* | *MUC1* | *PRDM1* | *SMAD3* | *VEGFB* |  |
| *BCL6* | *CYP19A1* | *FGF23* | *INHBA* | *MUTYH* | *PRKAA1* | *SMAD4* | *VEZF1* |  |
| *BCOR* | *CYP2C8* | *FGF3* | *IRF4* | *MYC* | *PRKAR1A* | *SMARCA1* | *VHL* |  |
| *BCORL1* | *CYP2D6* | *FGF4* | *IRS2* | *MYCL1/MYCL* | *PRKCA* | *SMARCA4* | *WHSC1L1* |  |
| *BCR* | *CYP3A* | *FGF6* | *ITGB2* | *MYCN* | *PRKCB* | *SMARCB1* | *WISP3* |  |
| *BLM* | *CYP3A4* | *FGF7* | *JAK1* | *MYD88* | *PRKCG* | *SMARCD1* | *WT1* |  |
| *BMPR1A* | *CYP3A5* | *FGFR1* | *JAK2* | *NAV3* | *PRSS8* | *SMC1A* | *WWP1* |  |
| *BRAF* | *DAXX* | *FGFR2* | *JAK3* | *NBN* | *PSMB1* | *SMC3* | *XIAP* |  |
| *BRCA1* | *DDR1* | *FGFR3* | *JUB(AJUBA)* | *NCOA1* | *PSMB2* | *SMO* | *XPA* |  |
| *BRCA2* | *DDR2* | *FGFR4* | *JUN* | *NCOA2* | *PSMB5* | *SOCS1* | *XPC* |  |
| *BRIP1* | *DIS3* | *FH* | *KAT6A* | *NCOR1* | *PTCH1* | *SOX10* | *XPO1* |  |
| *BTG1* | *DNMT1* | *FLCN* | *KDM5A* | *NEK11* | *PTCH2* | *SOX17* | *XRCC3* |  |
| *BTK* | *DNMT3A* | *FLT1* | *KDM5C* | *NF1* | *PTEN* | *SOX2* | *YES1* |  |
| *C11orf30* | *DOT1L* | *FLT3* | *KDM6A* | *NF2* | *PTP4A3* | *SOX9* | *ZNF217* |  |
| *C17orf39/GID4* | *DUSP6* | *FLT4* | *KDR* | *NFE2L2* | *PTPN11* | *SPEN* | *ZNF703* |  |
| *C1QA* | *EDNRA* | *FNTA* | *KEAP1* | *NFE2L3* | *PTPRD* | *SPOP* | *ZRSR2* |  |
| *C1R* | *EGFR* | *FOXA1* | *KIAA1549* | *NFKBIA* | *RAC2* | *SPRY4* |  |  |
| *C1S* | *EGR3* | *FOXA2* | *KIF1B* | *NKX2-1* | *RAD21* | *SRC* |  |  |
| *CARD11* | *EIF4A2* | *FOXL2* | *KIF5B* | *NKX3-1* | *RAD50* | *SRD5A2* |  |  |
| *CASP8* | *ELAC2* | *FPGS* | *KIT* | *NOTCH1* | *RAD51* | *SRSF2* |  |  |
| *CBFB* | *ELF3* | *FUBP1* | *KLC3* | *NOTCH2* | *RAD51B* | *SSTR2* |  |  |
| *CBL* | *EML4* | *FYN* | *KLF4* | *NOTCH3* | *RAD51C* | *SSTR5* |  |  |
| *CBLB* | *EP300* | *GAB2* | *KLHL6* | *NOTCH4* | *RAD51D* | *STAG2* |  |  |
| **OrigiMed Qiyuan ctDNA panel (329 genes)** | | | | | | | |  |
| *ABL1* | *CBL* | *DAXX* | *FLT4* | *KRAS* | *NR4A3* | *RAC1* | *SPOP* |  |
| *ABL2* | *CCND1* | *DDR2* | *FOXL2* | *LMO1* | *NRAS* | *RAD50* | *SPTA1* |  |
| *ACVR1B* | *CCND2* | *DICER1* | *FOXP1* | *LRP1* | *NRG1* | *RAD51* | *SRC* |  |
| *ACVR2A* | *CCND3* | *DNMT3A* | *FUBP1* | *LRP1B* | *NRG3* | *RAD51C* | *SRSF2* |  |
| *AKT1* | *CCNE1* | *DOT1L* | *FUS* | *LZTR1* | *NSD1* | *RAF1* | *SSX1* |  |
| *AKT2* | *CD274(PD-L1)* | *DPYD* | *FYN* | *MAP2K1(MEK1)* | *NSD2* | *RANBP2* | *STAG2* |  |
| *AKT3* | *CD79A* | *EGF* | *GATA1* | *MAP2K2(MEK2)* | *NTRK1* | *RARA* | *STAT3* |  |
| *ALK* | *CD79B* | *EGFR* | *GATA2* | *MAP2K4* | *NTRK2* | *RB1* | *STAT4* |  |
| *AMER1* | *CDC73* | *EP300* | *GATA3* | *MAP3K1* | *NTRK3* | *RBM10* | *STK11* |  |
| *APC* | *CDH1* | *EPHA3* | *GATA4* | *MAP3K13* | *NUP93* | *RECQL* | *SUFU* |  |
| *APOBEC3B* | *CDK12* | *EPHA5* | *GLI1* | *MCL1* | *PALB2* | *RET* | *SYK* |  |
| *AR* | *CDK4* | *EPHA7* | *GLI3* | *MDM2* | *PARK2* | *RHOA* | *TBX3* |  |
| *ARAF* | *CDK6* | *EPHB1* | *GNA11* | *MDM4* | *PARP1* | *RICTOR* | *TCF7L2* |  |
| *ARID1A* | *CDK8* | *ERBB2* | *GNAQ* | *MED12* | *PARP4* | *RNF43* | *TERT* |  |
| *ARID1B* | *CDKN1A* | *ERBB3* | *GNAS* | *MEN1* | *PAX5* | *ROCK1* | *TET1* |  |
| *ARID2* | *CDKN1B* | *ERBB4* | *GRIN2A* | *MET* | *PBRM1* | *ROCK2* | *TET2* |  |
| *ASXL1* | *CDKN2A* | *ERCC1* | *H3F3A* | *MGMT* | *PDCD1(PD-1)* | *ROS1* | *TFE3* |  |
| *ATM* | *CDKN2B* | *ERRFI1* | *HDAC9* | *MLH1* | *PDCD1LG2* | *RPTOR* | *TGFBR1* |  |
| *ATR* | *CDKN2C* | *ESR1* | *HGF* | *MPL* | *PDGFRA* | *RUNX1* | *TGFBR2* |  |
| *ATRX* | *CEBPA* | *ETV6* | *HNF1A* | *MRE11* | *PDGFRB* | *RUNX1T1* | *TOP1* |  |
| *AXIN1* | *CFTR* | *EZH2* | *HRAS* | *MSH2* | *PIK3CA* | *SDHA* | *TOP2A* |  |
| *AXIN2* | *CHD2* | *FAM135B* | *HSP90AA1* | *MSH6* | *PIK3CB* | *SDHB* | *TP53* |  |
| *AXL* | *CHD4* | *FAM46C* | *IDH1* | *MTOR* | *PIK3CD* | *SDHC* | *TP63* |  |
| *B2M* | *CHEK1* | *FANCA* | *IDH2* | *MUTYH* | *PIK3CG* | *SDHD* | *TSC1* |  |
| *BAP1* | *CHEK2* | *FANCC* | *IGF1R* | *MYC* | *PIK3R1* | *SETBP1* | *TSC2* |  |
| *BARD1* | *CIC* | *FANCD2* | *IKZF1* | *MYCL* | *PIK3R2* | *SETD2* | *TSHR* |  |
| *BCL2* | *COL1A1* | *FANCE* | *IL7R* | *MYCN* | *PMS2* | *SF3B1* | *U2AF1* |  |
| *BCL2L11* | *CRBN* | *FANCG* | *INPP4B* | *MYD88* | *POLB* | *SIK1* | *VEGFA* |  |
| *BCL6* | *CREB3L1* | *FANCM* | *ITK* | *NBN* | *POLD1* | *SLIT2* | *VHL* |  |
| *BCOR* | *CREBBP* | *FAS* | *JAK1* | *NCOA2* | *POLE* | *SMAD2* | *WEE1* |  |
| *BCORL1* | *CRKL* | *FAT1* | *JAK2* | *NCOR1* | *PPP2R1A* | *SMAD3* | *WEE2* |  |
| *BCR* | *CRLF2* | *FAT3* | *JAK3* | *NEK11* | *PRDM1* | *SMAD4* | *WT1* |  |
| *BLM* | *CSF1R* | *FAT4* | *JUN* | *NF1* | *PREX2* | *SMARCA4* | *XPO1* |  |
| *BMPR1A* | *CSK* | *FBXW7* | *KDM5A* | *NF2* | *PRKACA* | *SMARCB1* | *XRCC3* |  |
| *BRAF* | *CSNK1A1* | *FGFR1* | *KDM5C* | *NFE2L2* | *PRKCI* | *SMO* | *ZNF750* |  |
| *BRCA1* | *CTCF* | *FGFR2* | *KDM6A* | *NFIB* | *PRKDC* | *SNCAIP* |  |  |
| *BRCA2* | *CTNNA1* | *FGFR3* | *KDR* | *NFKBIA* | *PRSS1* | *SND1* |  |  |
| *BRD4* | *CTNNB1* | *FGFR4* | *KEAP1* | *NOTCH1* | *PRSS8* | *SOCS1* |  |  |
| *BRIP1* | *CUL3* | *FH* | *KIT* | *NOTCH2* | *PTCH1* | *SOX2* |  |  |
| *CAMTA1* | *CXCR4* | *FLCN* | *KMT2A* | *NOTCH3* | *PTEN* | *SOX9* |  |  |
| *CARD11* | *CYLD* | *FLT1* | *KMT2C* | *NOTCH4* | *PTPN11* | *SPEN* |  |  |
| *CASP8* | *CYP2D6* | *FLT3* | *KMT2D* | *NPM1* | *QKI* | *SPINK1* |  |  |
